# Polymorphisms of BCL2 and BAX Genes Associate with Outcomes in Advanced Non-small cell lung cancer Patients treated with platinum-based Chemotherapy

**DOI:** 10.1038/srep17766

**Published:** 2015-12-10

**Authors:** Yu Peng, Linang Wang, Yi Qing, Chongyi Li, Tao Ren, Qing Li, Mengxia Li, Shiheng Zhang, Jinglu Shan, Ge Wang, Zhenzhou Yang, Dong Wang

**Affiliations:** 1 Cancer Center, Daping Hospital and Research Institute of Surgery, The Third Military Medical University, Chongqing, China; 2 Department of Rehabilitation Medicine, Center of Bone Metabolism and Repair, State Key Laboratory of Trauma, Burns and Combined Injury, Trauma Center, Research Institute of Surgery, Daping Hospital, Third Military Medical University, Chongqing, China

## Abstract

Single-nucleotide polymorphisms (SNP) of the gene belonging to the BCL2 family are thought to play a role in chemotherapy resistance. This study investigated the association of BCL2-938C>A(rs2279115) and BAX-248G>A(rs4645878) promoter region SNPs and the clinical responses and outcomes of 235 non-small cell lung cancer (NSCLC) patients treated with platinum-based chemotherapy. The data suggested that BAX-248GA and GA+AA genotype was associated with poor response [odds ratio (OR) 1.943, p = 0.039; OR 1.867, p = 0.038, respectively] to chemotherapy, and BCL2-938CA, CA+AA and BAX-248GA, AA and GA+AA were associated with poor progression-free survival (PFS) [hazard ratio (HR) 1.514, p = 0.004; HR 1.456, p = 0.009; HR 1.449, p = 0.013; HR 2.006, p = 0.010; HR 1.506, p = 0.003, respectively] and BCL2-938CA, AA and CA+AA and BAX-248GA, AA and GA+AA were associated with poor overall survival (OS) (HR 2.006, p < 0.001; HR 2.322, p < 0.001; HR 2.096, p < 0.001; HR 1.632, p = 0.001; HR 2.014, p = 0.010; HR 1.506, p < 0.001, respectively). Furthermore, combination of these two polymorphisms showed patients with 2–4 variant alleles of these two genes associated with poor PFS and OS (HR 1.637, p = 0.001; HR 2.365, p < 0.001). The data from the current study provide evidence that BCL2-938C>A and BAX-248G>A polymorphisms may be useful in predicting clinical outcomes of patients with advanced inoperable NSCLC to platinum-based chemotherapy.

Lung cancer is a major cause of cancer-related mortality worldwide^1^. Histologically, lung cancer is usually classified as small cell or non-small cell lung cancer (NSCLC) and the latter represents up to 85% of all lung cancer cases and frequently is diagnosed at the later stages of disease, preventing curative surgery. Platinum-based chemotherapy is the first line standard treatment for NSCLC patients with advanced disease^2^; however, such treatment is often associated with poor response due to drug resistance. Chemotherapy resistance of NSCLC to platinum-based treatments is complex, but single-nucleotide polymorphisms (SNP) in apoptosis genes, particularly the *BCL2* family, may play a critical role^3^.

Platinum-based chemotherapeutic agents bind to and cause crosslinking of genomic DNA, especially in fast growing tumor cells, and trigger tumor cells to undergo apoptosis^4^. Apoptosis is a process of programmed cell death that occurs under both physiological and pathological conditions. Apoptosis regulates homeostasis in the human body. The balance of anti-apoptotic and pro-apoptotic proteins determines cell fate and regulates the response to apoptotic signals^5^,^6^,^7^. Deficiency in apoptosis alters intracellular homeostasis and may lead to carcinogenesis and promote tumor progression^8^. Two well-characterized regulators of apoptosis are the anti-apoptotic B-cell lymphoma 2 (*BCL2*) and the pro-apoptotic B-cell lymphoma 2-associated X protein (*BAX )*. BCL2 promotes cell survival by inhibiting apoptosis, whereas BAX promotes apoptosis^9^,^10^. Thus, aberrant expression of Bcl-2 and/or BAX is thought to play a role in cancer development. Additionally, SNPs in these genes have been reported to be associated with various human cancers such as head and neck squamous cell carcinoma^11^, endometrial cancer^12^, prostate cancer^13^, breast cancer^14^, acute lymphoblastic leukemia^15^ and glioma^16^. Furthermore, high *BCL2* expression has been associated with chemoresistance, and overexpression in cell lines has been observed to inhibit apoptosis^17^,^18^. Crosstalk occurs between chemotherapy-induced DNA damage and mitochondrial-induced apoptosis^19^. Previous studies have indicated that *BCL2* and *BAX* SNPs are associated with survival in various types of cancer^20^,^21^,^22^,^23^,^24^,^25^,^26^. Most studies of *BCL2* and *BAX* SNPs have focused on the promoter regions of these two genes, *BCL2*-938C>A (rs2279115) and *BAX*-248G>A(rs4645878), because they have been reported to be associated with altered expression of BCL2 and BAX^14^,^25^,^27^. The *BCL2*-938C>A A allele was associated with an increase in BCL-2 expression[14,27]. The *BAX*-248G>A A allele was associated with a decrease in BAX expression^25^. Hence, in this study, we hypothesized that *BCL2* and *BAX* polymorphisms, located in the untranslated promoter regions, could be associated with treatment responses and clinical outcomes in advanced NSCLC treated with platinum-based chemotherapy. To assess our hypothesis, we analyzed the responses and treatment outcomes of 235 patients with advanced NSCLC treated with platinum-based therapy and the association of treatment response and outcomes with *BCL2* -938C>A) (rs2279115) and *BAX* -248G>A (rs4645878) SNP status.

## Material and Methods

### Study Population, Response Assessment, Toxicity Evaluation and Survival Calculation

In this study, we enrolled a total of 235 inoperable NSCLC (stage III/IV) patients treated with at least two cycles of first line platinum-based chemotherapy between July 2007 and July 2012 from Daping Hospital, The Third Military Medical University (Chongqing, China). The study was approved by the ethics committee of the Daping Hospital and also carried out according to the protocols approved by the ethics committee. Only patients who understood the purpose of the study and signed the informed consent were included in the study. All patients had routine blood, hepatic and renal function tests, and an electrocardiogram. Patients had not received previous chemotherapy or radiotherapy and also had no other malignancies in the 5 years preceding this study. Patients were assessed for their Eastern Cooperative Oncology Group performance status (ECOG PS) and all participants were graded as level 0 ~ 2 before chemotherapy. Patients were treated with 75 mg/m^2^ cisplatin on day 1 plus 135 mg/m^2^ Taxol, 75 mg/m^2^ docetaxel, or 1000 mg/m^2^ gemcitabine on day 1 and day 8. The therapy cycles were repeated every 3–4 weeks.

Standard Response Evaluation Criteria in Solid Tumors (RECIST 1.0) were used to evaluate the treatment response, and the response was assessed by comparison of the baseline MRI or CT images with the follow-up images after every two cycles of chemotherapy. Patients were categorized as responders (complete response and partial response; CR or PR) or nonresponders (stable disease and progressive disease; SD or PD).

Chemotherapy-related toxicities were recorded for each treatment cycle, including leukocytopenia, anemia, thrombocytopenia, nausea, vomiting, diarrhea, neuropathy, weakness, hypersensitivity reaction, and renal toxic effects. Grade 3/4 toxicity (defined by the National Cancer Institute common toxicity criteria version 3.0) was assessed twice a week during chemotherapy.Progression-free survival (PFS) was calculated from the start of treatment to documentation of the first date of disease progression (death was considered a progression event in patients who died before disease progression). Overall survival (OS) was calculated from the start of treatment to death. Patients without documented death or objective progression at the time of the final analysis were censored at the time of their last objective tumor assessment or at the date they were last known to be alive. The survival data in this study were censored on July 23, 2014. Ten (4.3%) cases were censored.

### Genotyping

A 5 mL whole-blood sample was obtained from each patient before chemotherapy. Genotyping of *BCL2*-938C>A SNPs and *BAX*-248G>A was conducted by using the PCR-restriction fragment length polymorphism (PCR-RFLP) method. Each PCR amplification was performed in a 25 μl reaction mixture containing 12.5 pmol of each primer, 2 μl genomic DNA, 0.5 U of TaqMan SNP Genotyping Assay Mix (40x), 0.30 mM of dNTPs, 1.5 mM total MgCl_2_, and 5 μl of PCR buffer (5×).

The primers used to detect the *BCL2*-938C>A polymorphism were 5′-CTGCCTTCATTTATCCAGCA-3′ and 5′-GGCGGCAGATGAATTACAA-3′^11^. The PCR conditions consisted of an initial 96 °C for 5 min, 35 cycles at 96 °C for 45 s, 56 °C for 40 s and 72 °C for 30 s and a final extension at 72 °C for 10 min. The PCR products were then digested by BccI (New England BioLabs, Beverly, MA) overnight at 37 °C. The wild-type allele (CC) produced two bands of 189 and 111 bp; wild-type/variant allele (CA) produced three bands of 111, 189 and 300 bp, and the variant allele (AA) produced a single 300 bp band.

The primers for the *BAX*-248G>A polymorphism were 5′-CGGGGTTATCTCTTGGGC-3′ and 5′-GTGAGAGCCCCGCTGAAC-3′'^28^. The PCR conditions consisted of an initial denaturation at 95 °C for 5 min, followed by 40 cycles of 30 s at 94 °C, 30 s at 56 °C, and 45 s at 72 °C and a final extension at 72 °C for 5 min. The PCR products were then digested by Aci I (Aci I, which recognizes CCGC, New England BioLabs) at 37 °C for 15 min. Homozygous GG alleles (wild-type) were visualized as three major bands of 352, 256, and 96 base pair (bp) with the highest intensity for the 256-bp band under the UV light. In the heterozygous condition (AG alleles) the 256bp and 96 bp products are fainter, while the 352bp band is more intense. In contrast, the homozygous AA alleles (homozygous carrier of this SNP) showed only one 352-bp band.

### Statistical Analyses

Statistical analyses were performed using SPSS (IBM SPSS Statistics 19, SPSS, Chicago, IL, USA). Differences in response to treatment or grade 3/4 toxicities were analyzed between groups with different genotypes of these SNPs (variants vs. the wild-type) by calculation of *p* values using the Pearson χ2 test or the Fisher exact test. Multivariate logistic regression analyses were used to assess associations between the selected SNPs and chemotherapy response or toxicity by estimating odds ratios (ORs) and 95% confidence intervals (CIs). Survival curves for PFS and OS were determined using the Kaplan-Meier method and the Log-rank test was used to generate p values. Multivariate Cox proportional hazards models (Forward Stepwise: Likelihood Ratio) were used to estimate adjusted hazard ratios (HR) with 95% confidence intervals. Multivariable regressions analysis was adjusted for age, gender, ECOG stage, histology, disease stage, smoking status, weight loss, and chemotherapy regimens. Two-sided *p*-values of less than 0.05 were considered statistically significant. In order to adjust the p-value due to multiple comparisons, the Bonferroni-Holm method was used to calculate the *p*-value for the results of any SNP.

## Results

### Patient characteristics

Patients’ characteristics and genotypic data are shown in Table 1. In brief, all patients were Chinese with 180 (76.6%) males and 55 (23.4%) females and a median age of 58 years old (range between 29 and 84 years old). Histologically, 133 of these 235 patients were adenocarcinoma, while 78 were squamous cell carcinoma and 24 were other types of NSCLC; 43 (18.3%) patients had clinical stage IIIA disease, 38 (16.2%) stage IIIB and 154 (65.5%) stage IV disease. Furthermore, 124 (52.8%) patients had ECOG PS 0 and 111 (47.2%) patients had ECOG PS ≥ 1 and 91 (38.7%) never smoked tobacco and 76 (32.3%) patients had weight loss before chemotherapy. *BCL2-*938C>G, 88 (37.4%) patients were CC genotype, 104 (44.3%) were CG genotype and 43 (18.3%) were GG genotype. *BAX*-248G>A, 129 (54.9%) patients were GG genotype, 87 (37.0%) were GA genotype and 19 (8.1%) were AA genotype. A significant association was found with histology (p = 0.005) between the *BCL2*–938C>A genotype and clinicopathologic factors (Table 2).

For chemotherapy, 180 (76.6%) patients received TP regimens (Taxol/docetaxel plus cisplatin), 32 (13.6%) had GP regimens (gemcitabine plus cisplatin), and 23 (9.80%) had other platinum combinations (vinorelbine plus cisplatin or pemetrexed plus cisplatin). The median number of chemotherapy cycles was four (range between 2 and 8).

### Association of Genotypes with Treatment Response and Toxicity

The overall response rate of patients to platinum-based chemotherapy was 35.7% with the following responses to the first-line treatments: one patient had CR (0.4% of evaluable patients), 83 PR (35.32%), 75 SD (31.9%), and 76 PD (32.3%). A numerically increased rate of response was observed in patients with the *BAX*-248GG vs. the GA or GA+AA phenotype but the differences did not reach statistical significance after multiplicity adjustment (42.6% vs. 27.6%, *p* = 0.024; 42.6% vs. 27.4%, *p* = 0.015). No statistically significant association was observed between BCL2-938C>A and responses to chemotherapy (Table 3). After adjusting for age, gender, ECOG PS, histology, tumor stage, smoking status, chemotherapy regimens, weight loss, *BCL2*-938C>A, and *BAX*-248G>A, the logistic regression model analysis showed that the following parameters were able to predict the treatment response: ECOG PS (adjusted OR 2.112, 95% CI 1.156–3.857, *p* = 0.015), tumor stage (adjusted OR 2.887, 95% CI 1.510–5.518, *p* = 0.001), smoking status (adjusted OR 2.446, 95% CI 1.140–5.247, *p* = 0.022) and *BAX*-248G>A (GG vs. GA, adjusted OR 1.943, 95% CI 1.035–3.648, *p* = 0.039; GG vs. GA+AA, adjusted OR 1.867, 95% CI 1.035–3.369, *p* = 0.038).

The incidence of grade 3/4 toxicity was 57.5%. In particular, 90 patients (38.3%) had grade 3/4 hematologic toxicity, 44 patients (18.7%) had grade 3/4 gastrointestinal toxicity, 27 patients (11.49%) had both and 127 patients (54.04%) had neither hematologic toxicity nor gastrointestinal toxicity. Our SNP data showed that there was no significant association between risk of grade 3/4 hematologic toxicity or gastrointestinal toxicity and BCL2-938C>A (adjusted OR 1.006, 95% CI 0.680 ~ 1.490, *p* = 0.975; adjusted OR 0.927, 95% CI 0.572 ~ 1.500, *p* = 0.756) or *BAX*-248G>A (adjusted OR 0.726, 95% CI 0.464 ~ 1.490, *p* = 0.160; adjusted OR 0.976, 95% CI 0.576 ~ 1.655, *p* = 0.929) polymorphisms (Logistic regression model analysis). However, chemotherapeutic regimens (TP vs. GP, adjusted OR 2.566, 95% CI 1.154 ~ 5.706, *p* = 0.021) and age (adjusted OR 1.802, 95% CI 1.030 ~ 3.152, *p* = 0.039) were associated with the risk of hematologic toxicity (Logistic regression model analysis).

### Association of Genotypes with PFS and OS of these patients after chemotherapy

The median follow-up time was 22 months (range between 1.8 and 70.4 months) and 214 (91.06%) patients died during the follow-up. The one-year survival rate was 60% and the 2-year survival rate was 25%. The median PFS was 7.0 months (95% CI, 6.058–7.942). The median OS was 15.0 months (95% CI, 12.981–17.019).

**Figure 1:**
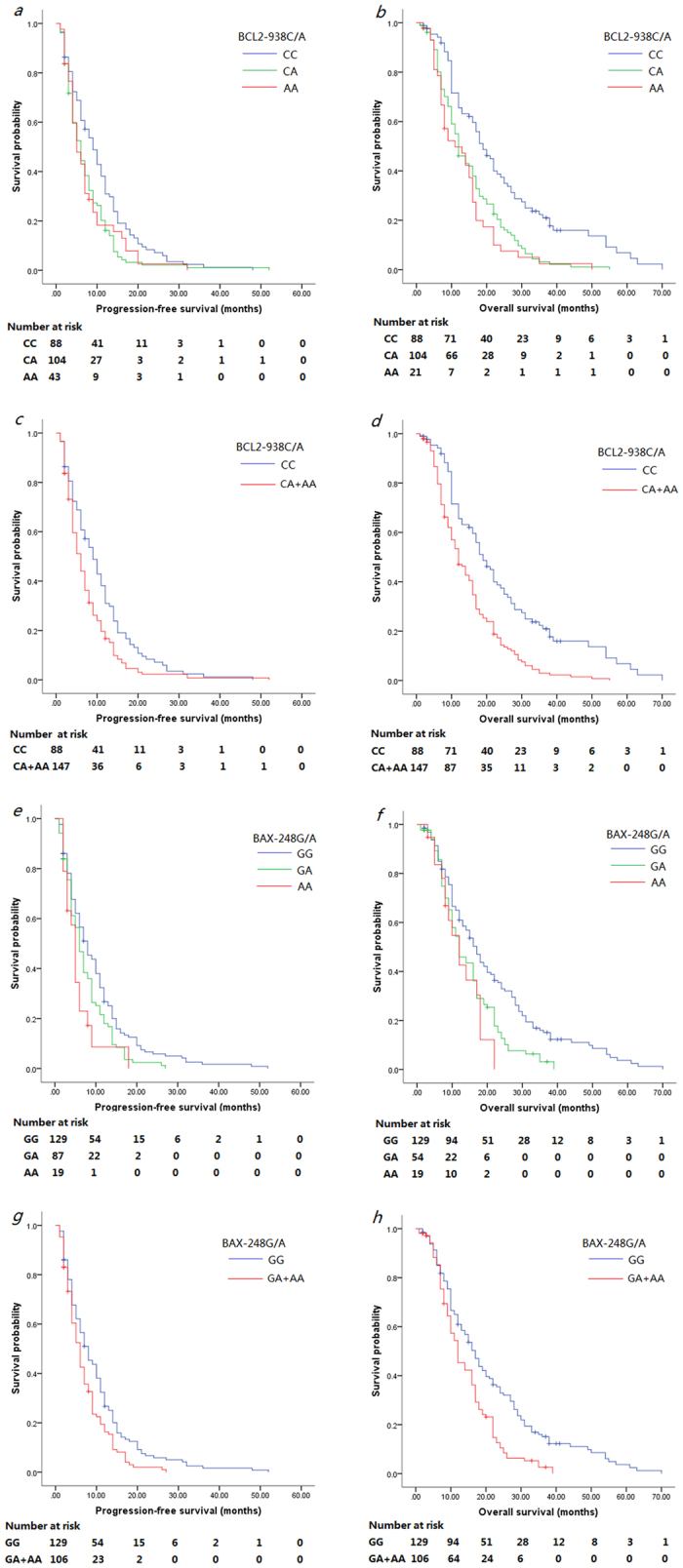
Association of *BCL2* and *BAX* SNPs with survival of NSCLC patients. (**a**–**d**) Kaplan-Meier curves of PFS and OS stratified by patients with different *BCL2-*938C>A genotypes. (**e**–**h**) Kaplan-Meier curves of PFS and OS stratified by patients with different *BAX-*248G>A genotypes.

In univariate analysis, patients with *BCL2*-938AA, CA or CA+AA genotype had significantly shorter median PFS compared with those with −938CC genotype [9 m vs. 5 m, *p* = 0.035 (the difference did not reach statistical significance after multiplicity adjustment); 9 m vs. 6 m, *p* = 0.004; 9 m vs. 6 m, *p* = 0.003; Log-rank test; respectively; Fig. 1a,c. tif]. The median PFS of patients with the *BAX*-248GG genotype was significantly longer than those with the -248GA, AA or GA+AA genotype [8 m vs. 6 m, *p* = 0.018; 8 m vs. 5 m, *p* = 0.010; 8 m vs. 6 m, *p* = 0.004 (significance remained after the Bonferroni–Holm method); Log-rank test; respectively; Fig. 1e,g. tif]. The multivariate analysis showed that age (HR 0.638, 95% CI 0.480–0.846, *p* = 0.002), ECOG PS (HR 1.906, 95% CI 1.432–2.537, *p* < 0.001), *BCL2*–938C>A (CC vs. CA, HR 1.570, 95% CI 1.152–2.140, *p* = 0.004; CC vs. CA+AA, HR 1.456,95% CI 1.099–1.929, *p* = 0.009) and *BAX*–248G>A (GG vs. GA, HR 1.449, 95% CI 1.080–1.945, *p* = 0.013; GG vs. AA, HR 2.006, 95% CI 1.184–3.399, *p* = 0.010; GG vs. GA+AA, HR 1.506, 95% CI 1.145–1.980, *p* = 0.003) were all significant prognostic indictors for PFS (Table 4).

Furthermore, in the univariate model (Table 4), the *BCL2*-938C>A polymorphism was significantly associated with OS, i.e., patients carrying the CC genotype had a longer median OS than patients carrying the CA, AA or CA+AA genotype (19 m vs. 12 m, *p* < 0.001; 19 m vs. 11 m, *p* < 0.001; 19 m vs. 12 m, *p* < 0.001; Log-rank test; respectively; Fig. 1b,d. tif). The median survival time of patients with *BAX*-248GG genotypes was significantly longer than those with the -248GA, AA or GA+AA genotype (17 m vs. 12 m, *p* = 0.001; 17 m vs. 12 m, *p* = 0.005; 17 m vs. 12 m, *p* < 0.001; Log-rank test; respectively; Fig. 1f,h. tif). The multivariate analysis showed that ECOG PS (HR 2.430, 95% CI 1.823–3.238, *p* < 0.001), *BCL2*-938C>A (CC vs. CA, HR 2.006, 95% CI 1.462–2.752, *p* < 0.001; CC vs. AA, HR 2.322, 95% CI 1.558–3.461, *p* < 0.001; CC vs. CA+AA, HR 2.096, 95% CI 1.555–2.824; *p* < 0.001), and *BAX*-248G>A (GG vs. GA, HR 1.632, 95% CI 1.210–2.199, *p* = 0.001; GG vs. AA, HR 2.014, 95% CI 1.188–3.425, *p* = 0.010; GG vs. GA+AA, HR 1.705, 95% CI 1.283–2.266, *p* < 0.001) were all independent predictors for OS of these NSCLC patients.

**Figure 2:**
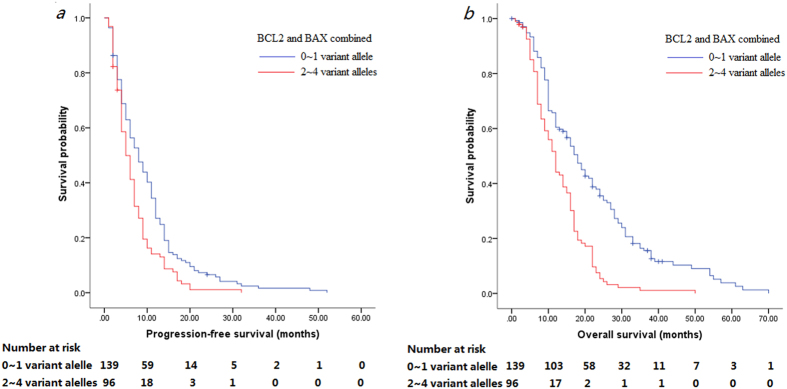
Kaplan-Meier curves of PFS (**a**) and OS (**b**) stratified by patients with different numbers of risk alleles of *BCL2-*938C>A and BAX-248G>A polymorphisms.

After that, we combined these two BCL2-938C>A and BAX-248G>A polymorphisms for association with PFS and OS of patients. Our data showed that the -938A and -248A were classified as adverse alleles based on association with higher risk of progression and death presented in Table 4 and as the number of adverse allele increased, the median PFS and OS decreased. Patients with more than 2 adverse alleles had shorter median PFS (5 m vs. 8 m, *p* = 0.001) and OS (12 m vs. 18 m, *p* < 0.001) compared to those with 0–1 adverse alleles (Table 4, Fig. 2. tif). The multivariate analysis showed that patients with 2–4 adverse alleles had an increased risk of disease progression (HR 1.637, 95% CI 1.240 ~ 2.161, *p* = 0.001) and death (HR 2.365, 95% CI 1.760–3.178, *p* < 0.001). These significances remained after the Bonferroni-Holm method analysis.

## Discussion

Cisplatin resistance occurs through a variety of mechanisms, such as changes in cellular uptake and efflux of the drug, increased expression of detoxification enzymes, increased DNA repair or inhibition of apoptosis^29^. In this study, we investigated whether the occurrence of SNPs, located in the promoter regions of two apoptosis-related genes are associated with responses and/or outcomes in patients with advanced NSCLC that are treated with cisplatin-based chemotherapy. A numerically increased rate of response was observed in patients with the *BAX*-248GG vs. the GA or GA+AA phenotype but the difference did not reach statistical significance after multiplicity adjustment. We found no significant association between the *BCL2*-938C>A polymorphism and chemotherapy response. There was no statistical association between *BCL2*-938C/A or *BAX*-248G>A polymorphism and grade 3/4 hematologic or gastrointestinal toxicity in these patients. Furthermore, patients with the *BCL2*-938C>A variant genotype (A allele) or *BAX*-248G>A variant genotype (A allele) associated with poor PFS and OS. The combined *BCL2*-938C>A and *BAX*-248G>A were also associated with PFS and OS of the patients. The multivariate analysis showed that ECOG PS, *BCL2*-938C>A, and *BAX*-248G>A were all independent predictors for OS of these NSCLC patients. To the best of our knowledge, this is the first study of this kind to demonstrate an association between the *BAX*-248G>A or the combination of *BCL2*-938C>A and *BAX*-248G>A with outcome of advanced NSCLC patients to cisplatin-based chemotherapy.

*BCL2* is localized to chromosome 18q21.3^30^, coding a protein with three exons and two gene promoters (P1 and P2). These two promoters have different functions in regulation of BCL2 expression, i.e., the P2 promoter is localized to the translation initiation site and functions as a negative regulatory element of the P1 promoter^31^,^32^. Park *et al.*^33^ identified BCL2-938C>A(rs2279115) in P2 and Nückel *et al.*^27^ found that the -938C allele of BCL2-938C>A displayed a significant increase in *BCL2* promoter activity. Concomitantly, expression of BCL2 protein in B-lymphocytes from chronic lymphocytic leukemia patients carrying the -938 AA genotype was significantly increased compared with CC genotypes. This has been confirmed by other studies of prostate cancer^34^, renal cancer^24^, oropharyngeal squamous cell carcinoma^35^ and breast cancer^36^, although Zhang *et al.*^14^ have an opposite result showing that the −938A allele contributed to decreased expression of the *BCL2* protein in breast cancer cell lines. Furthermore, previous studies showed that the *BCL2*-938 A variant was associated with a decreased risk of head and neck squamous cell carcinoma^11^ and prostate cancer^13^, but increased risk of breast cancer^14^ and glioma^16^. The –938 AA genotype was independently associated with worse PFS and OS of prostate cancer^34^, chronic lymphocytic leukemia^27^, glioblastoma multiforme^21^, and NSCLC^20^, whereas it associated with a better prognosis of lymph node-negative invasive breast cancer^36^, oropharyngeal squamous cell carcinoma^35^, renal cancer^24^, ovarian cancer^37^, and limited-disease small cell lung cancer^38^. In our current study, we found that the *BCL2*-938 A variant was associated with poor PFS and OS of patients with advanced NSCLC after chemotherapy, which supports previous data reported by Katsuhiro Masago and colleagues of 168 advanced NSCLC patients who received platinum-based chemotherapy^20^. Expression of the anti-apoptotic BCL2 protein should contribute to resistance to cisplatin-induced tumor cell apoptosis and thus lead to a poor response and outcome. Indeed, BCL2 overexpression has been shown to be a marker of chemotherapy resistance in both SCLC and NSCLC^39^,^40^. However, Fontanini *et al.*^41^ reported that survival probability was higher in patients with BCL2-expressing, resected NSCLC because of the less aggressive behavior of NSCLC with BCL2 overexpression. A meta-analysis of 28 studies revealed that BCL2 overexpression had a positive influence on survival of NSCLC^42^. However, further study is needed to clarify this discrepancy.

*BAX* is localized to chromosome 19q13.3, coding a protein with six exons and a promoter^43^. The *BAX* promoter binds to different transcription factors or proteins, such as p53 response elements, the TATA box, canonical E-boxes, and the NF-κB binding site to regulate BAX expression^44^. *BAX* was extensively studied in different types of cancer such as pancreatic cancer^45^, colon cancer^46^,^47^, esophageal cancer^48^, lung cancer^49^,^50^, squamous cell carcinoma of the head and neck^11^, prostate carcinoma^51^, ovarian carcinoma^52^, and breast cancer^53^. Recently, BAX-248G>A (rs4645878) was reported to be associated with reduced expression of BAX protein and altered susceptibility to chronic lymphocytic leukemia^25^,^26^, although a meta-analysis of seven independent studies with 1772 cases and 1708 controls revealed that neither allele frequency nor genotype of BAX-248G>A associated with risk of human cancer using different genetic models^54^. Several studies reported that patients with BAX low expression had a significantly longer median survival in NSCLC^50^, esophageal squamous cell carcinoma^48^, and colon cancer^47^. Moreover, Paola Perego *et al.*^55^ showed that *p53* mutations developed cisplatin resistance in ovarian cancer as a consequence of the loss of p53 transactivation of BAX expression. Our current SNP data further confirm the role of BAX in the regulation of cisplatin resistance and shorter PFS and OS of patients with advanced NSCLC.

Indeed, previous studies showed that *BAX* was able to heterodimerize with BCL2 and Mcl-1 and that overexpression of BCL2 and Mcl-1 proteins compromised the proapoptotic capacity of BAX^56^,^57^. Therefore, we determined to assess whether BCL2 and BAX interact synergistically to contribute to cisplatin resistance and alter the PFS and OS of patients with advanced NSCLC. We found that as the number of variant alleles increased, the median PFS and OS were decreased accordingly. Patients with more than 2 variant alleles had a much shorter median PFS and OS compared to those carrying 0–1 variant alleles.

Our current study is just the first of this kind for proof-of-principle. Although we showed that the BCL2-938C>A and BAX-248G>A SNPs significantly associated with platinum-based chemotherapy response, PFS and OS of patients with advanced NSCLC, we did not show the association of these SNPs with chemotherapy-related toxicities. However, the mechanism by which BCL2 SNPs may influence the clinical outcomes of patients with advanced NSCLC to platinum-based chemotherapy is unclear. This raises the question of whether a patient with such polymorphisms would have better survival than those without these polymorphisms in the absence of treatment. Do the polymorphisms actually affect the effectiveness of the regimen, or do they simply afford a better prognosis to patients? Hence, further work is necessary; for example, a larger sample size and a prospective study would be able to confirm our current data. Variation of BCL2 and BAX expression associates with an altered sensitivity and clinical outcome of NSCLC patients to chemotherapy.

## Additional Information

**How to cite this article**: Peng, Y. *et al.* Polymorphisms of BCL2 and BAX Genes Associate with Outcomes in Advanced Non-small cell lung cancer Patients treated with platinum-based Chemotherapy. *Sci. Rep.* **5**, 17766; doi: 10.1038/srep17766 (2015).

## Figures and Tables

**Table 1:** Patients’ characteristics (n = 235).

Characteristics	n	%
Age, years		
Median	58	
Range	29–84	
Gender		
Male	180	76.6
Female	55	23.4
Stage		
IIIA	43	18.3
IIIB	38	16.2
IV	154	65.5
Histologic type		
Adenocarcinoma	133	56.6
Squamous cell	78	33.2
Others^1^	24	10.2
Smoking history		
Never smoke	91	38.7
Ever smoke	144	61.3
ECOG PS		
0	124	52.8
1	90	38.3
2	21	8.9
Weight loss		
Yes	76	32.3
No	159	67.7
Chemotherapy regimens		
Platinum-paclitaxel	180	76.6
Platinum-gemcitabine	32	13.6
Other platinum combinations^2^	23	9.8
Response		
CR	1	0.4
PR	83	35.3
SD	75	31.9
PD	76	32.3
Toxicity		
Grade 3/4 hematologic toxicity	91	38.7
Grade 3/4 gastrointestinal toxicity	44	18.7
*BCL2*-938C>A		
CC	88	37.4
CA	104	44.3
AA	43	18.3
*BAX*-248G>A		
GG	129	54.9
GA	87	37.0
AA	19	8.1

Abbreviations: ECOG PS, Eastern Cooperative Oncology Group performance status; TNM, tumor-node-metastasis; CR, complete response; PR, partial response; SD, stable disease; PD, progressive disease.

^1^Others include mixed cell, neuroendocrine carcinoma, or undifferentiated carcinoma.

^2^Other platinum combinations: vinorelbine plus cisplatin or pemetrexed plus cisplatin.

**Table 2:** Association between the *BCL2*-938C>A/*BAX*-248G>A genotype and clinicopathologic factors.

Characteristics	*BCL2*-938C>A			P^1^	BAX-248G>A			P^1^
	CC	CA	AA		GG	GA	AA	
Gender								
Male	67	83	30		100	64	16	
Female	21	21	13	0.422	29	23	3	0.558^2^
Smoking history								
Never smoke	38	35	18		53	34	4	
Ever smoke	50	69	25	0.360	76	53	15	0.220^2^
Histologic type								
Adenocarcinoma	41	59	33		73	47	13	
Non-adenocarcinoma	47	45	10	0.005	56	40	6	0.518

^1^Pearson χ2 test.

^2^Fisher exact test.

**Table 3:** Association of these two gene genotypes with treatment response and toxicity.

Genotype	N, %	Treatment response^1^			Toxicity^1^					
		CR+PR	SD+PD	*P*	Hematologic toxicity		*p*	Gastrointestinal toxicity		*p*
		n (%)	n (%)		G0-2	G3-4		G0-2	G3-4	
BCL2-938C>A										
CC	88	33 (37.5)	55 (62.5)		54 (61.4)	34 (38.6)		72 (81.8)	16 (18.2)	
CA	104	38 (36.5)	66 (63.5)	0.891	63 (60.6)	41 (39.4)	0.911	81 (77.9)	23 (22.1)	0.500
AA	43	13 (30.2)	30 (69.8)	0.413	28 (65.1)	15 (34.9)	0.677	37 (86.0)	6 (14.0)	0.543
CA+AA	147	51 (34.7)	96 (65.3)	0.664	89 (60.5)	56 (39.5)	0.998	118 (80.3)	29 (19.7)	0.771
BAX-248G>A										
GG	129	55 (42.6)	74 (57.4)		76 (58.9)	53 (41.1)		105 (81.4)	24 (18.6)	
GA	87	24 (27.6)	63 (72.4)	0.024^3^	54 (62.1)	33 (37.9)	0.642	69 (79.3)	18 (20.7)	0.704
AA	19	5 (26.3)	14 (72.7)	0.176	15 (78.9)	4 (21.1)	0.130^2^	16 (84.2)	3 (15.8)	0.767
GA+AA	106	29 (27.4)	77 (72.6)	0.015^3^	69 (65.1)	37 (34.9)	0.332	85 (80.2)	21 (19.8)	0.815

Abbreviations: CR, complete response; PR, partial response; SD, stable disease; PD, progressive disease.

^1^Pearson χ2 test.

^2^Fisher exact test.

^3^Significance didn’t remain after the Bonferroni–Holm method.

**Table 4:** Association of BCL2 and BAX polymorphisms with PFS and OS of NSCLC patients.

Genotype	N	PFS (months)					OS (months)					
		Median	*p*^1^	HR^2^	95% CI	*p*^2^	Median	*p*^1^	HR^2^	95% CI	*p*^2^	
BCL2-938C>A												
CC	88	9					19					
CA	104	6	0.004	1.570	1.152–2.140	0.004	12	<0.001	2.006	1.462–2.752	<0.001	
AA	43	5	0.035^3^	1.465	0.991–2.166	0.055	11	<0.001	2.322	1.558–3.461	<0.001	
CA+AA	147	6	0.003	1.456	1.099–1.929	0.009	12	<0.001	2.096	1.555–2.824	<0.001	
BAX-248G>A												
GG	129	8					17					
GA	87	6	0.018^3^	1.449	1.080–1.945	0.013	12	0.001	1.632	1.210–2.199	0.001	
AA	19	5	0.010^3^	2.006	1.184–3.399	0.010	12	0.005	2.014	1.188–3.425	0.010	
CA+AA	106	6	0.004	1.506	1.145–1.980	0.003	12	<0.001	1.705	1.283–2.266	<0.001	
BCL2 and BAX combined												
0 ~ 1 variant allele	139	8					18					
2 ~ 4 variant alleles	96	5	0.001	1.637	1.240–2.161	0.001	12	<0.001	2.365	1.760–3.178	<0.001	

Abbreviations: PFS, progression-free survival; OS, overall survival; HR, hazard ratio; CI, confidence interval.

^1^Log-rank test.

^2^Multivariate Cox proportional hazards models (Forward Stepwise: Likelihood ratio).

^3^Significance didn’t remain after the Bonferroni–Holm method.
